# The Effect of Short-Chain Fatty Acids on Growth of *Cryptosporidium parvum* In Vitro

**DOI:** 10.3390/microorganisms10091822

**Published:** 2022-09-11

**Authors:** Aidan P. Keelaghan, Raheela Charania, Jan R. Mead

**Affiliations:** 1 Department of Pediatrics, Emory University and Children’s Healthcare Organization of Atlanta, Atlanta, GA 30322, USA; 2 Atlanta VA Medical Center, Decatur, GA 30033, USA

**Keywords:** *Cryptosporidium*, microbiome, short-chain fatty acid, antibiotics, cloxacillin, apoptosis, histone deacetylase inhibitors, autophagy

## Abstract

In a previous study, we observed an increase in the severity of cryptosporidial infection corresponding to decreased levels of short-chain fatty acids (SCFAs). Therefore, we decided to examine the effect of SCFAs on *Cryptosporidium* growth in human ileocecal adenocarcinoma (HTC-8) cells. HTC-8 cells were infected with 1 × 10^5^ *C. parvum* oocysts. After 48 h of incubation with selected SCFAs, cells were fixed and labeled with monoclonal antibody directed to all intracellular stages, and the number of parasites was quantitated using a fluorescent microscope. Acetate, butyrate, propionate and valproate significantly inhibited growth, with an EC_50_ between 4 and 10 mM. Additionally, when combined, butyrate, acetate and propionate showed increased efficacy. Butyrate also inhibited growth when incubated with sporozoites prior to infection of host cell monolayers. In addition, we looked at possible mechanisms of action of inhibition. A combination of *C. parvum* infection and butyrate treatment led to increases in apoptosis and certain inflammatory cytokines. We conclude that acetate, propionate and butyrate have direct inhibitory activities in host cells against *C. parvum*, and butyrate can also affect sporozoite infectivity directly. While not preventing infection, SCFAs may help in keeping the infection low or in check.

## 1. Introduction

*Cryptosporidium* spp. are opportunistic protozoan parasites that infect epithelial cells of the small intestine, causing diarrheal illness in humans. While in immunocompetent individuals, the disease may be mild to moderate and usually self-limiting, cryptosporidiosis in immunocompromised individuals can be severe and life threatening [1]. Additionally, it is the second major cause of diarrheal illness in children globally [2], and repeated infections can lead to long-term growth deficits and cognitive impairment [3].

Differences in infection severity may be due to multiple factors, including immunological status of the host, malnutrition, prior exposure to the parasite or changes in host microbiota. While cryptosporidiosis is the result of *Cryptosporidium* colonizing the gut epithelium, an environment adjacent to trillions of bacteria, the impact of said microbiome on cryptosporidial infection is poorly understood. In one study, microbiota variations in the stools of immunocompetent human volunteers experimentally infected with *C. parvum* correlated with metabolite composition and differences in susceptibility to infection, implicating the importance of gut microflora in infection [4].

Resident bacterial taxa affect resistance to experimental cryptosporidial infections in mouse models. SCID mice with established conventional gut flora are more resistant to infection by *C. parvum* than germ-free SCID mice [5]. A TCR-α-deficient mouse model of cryptosporidiosis reportedly developed IBD-like lesions following infection [6]. Interestingly, TCR-α-deficient mice exhibited more severe mucosal lesions when infected germ-free versus mice bearing conventional gut flora [6], suggesting microbiome influences on the pathology of disease.

In previous work, we determined how differences in microbiota affect resistance and susceptibility to cryptosporidial infections [7]. *C. parvum* parasite loads increased after cloxacillin treatment, suggesting decreases in certain commensals (predominantly Gram positives) are important in maintaining resistance to infection and for maintaining a heathy gut environment since they generate many important metabolites [7]. In particular, certain short-chain fatty acids were found to be significantly reduced in cloxacillin-treated mice. Alternatively, *C. parvum* infections may alter the microbiota and thus metabolites such as short-chain fatty acids (SCFAs). In one report, the SCFA butyrate was depleted in *C. parvum*-infected goats [8].

While SCFAs are known to affect immune cells such as type 3 innate lymphoid cells (ILC3s), B cells and the regulating activity of regulatory T cells [9], they can also have a direct effect on host epithelial cells [10]. Potential mechanisms include gene expression, autophagy and inducing apoptosis [11]. In this study, we examined the effect of SCFAs on cryptosporidial growth in infected human ileocecal adenocarcinoma (HCT-8) cells and also potential mechanisms of action by which these SCFAs may facilitate host resistance.

## 2. Materials and Methods

### 2.1. Preparation of Compounds

Short-chain fatty acid compounds (Sigma-Aldrich Inc., St. Louis, MO, USA) were prepared as stock solutions (50 mM) in dimethyl sulfoxide and diluted to working concentrations in culture media for evaluation in our in vitro culture assay.

### 2.2. C. parvum In Vitro Culture Assay

Growth of *C. parvum* in cell culture was performed as described previously [12]. Briefly, purified *C. parvum* IOWA isolate oocysts were washed with phosphate-buffered saline (PBS, pH 7.4) to remove the storage buffer (2.5% *w*/*v* aqueous potassium dichromate) and resuspended in RPMI 1640 media supplemented with 0.75% sodium taurocholate and incubated for 30 min at 37 °C. The excystation mixture containing oocysts, oocyst shells and free sporozoites was diluted with RPMI 1640 with 10% fetal bovine serum (FBS). Approximately 1 × 10^5^ oocysts and sporozoites were dispensed per well in 24-well plates containing confluent HCT-8 cells. Host cells inoculated with the parasite mixture were incubated for 3 h and subsequently washed with PBS to remove free parasites (those that had not invaded host cells). PBS was replaced with fresh RPMI 1640 with 10% FBS with or without test compounds and the cultures were incubated for 48 h. In addition, RPMI 1640 with 10% FBS supplemented with DMSO (same concentration used with test compounds) was evaluated to determine the effect of inhibition or toxicity on cultures. Culture wells were washed with PBS, and then PBS was replaced by Bouin’s solution. After fixation of cultures, the Bouin solution was removed and decolorized with 70% ethanol, followed by 5 washes with PBS. Culture wells were labeled with an anti-*C. parvum* fluorescein-labeled monoclonal antibody (C3C3-FITC) and parasites were enumerated via quantitative fluorescent microscopy at 400× magnification using an Olympus IX73 microscope (Waltham, MA, USA). Twenty-four sequential, non-overlapping fields per well were captured as digital images, constituting a representative subsample of the entire well. The parasites were quantified using OpenCFU (https://sourceforge.net/projects/opencfu/, accessed on 4 October 2014) software [13] based on the size of the fluorescing parasites and the parasite-specific fluorescent signal. Data were obtained from four well replicates, and each experiment was repeated at least twice. Dose–effect curves and the median effective concentration (EC_50_) of each compound alone or in combination was determined using CompuSyn (ComboSyn, Inc., Paramus, NJ, USA, ver. 2.1 for Microsoft Windows) computer software. The EC_50_ was defined as the concentration required for reducing the number of parasites by 50% compared with the untreated control.

CompuSyn software was used in combination studies to calculate the effect of multiple short-chain fatty acids. The analyses were based on the Chou–Talalay method [14] for drug combinations, which calculates the combination index (CI). The CI is a quantitative representation of pharmacological interactivity of multiple drug combinations, where <1 is antagonistic, =1 is additive and >1 is synergistic.

### 2.3. Preparation of Live Sporozoites and SCFA Treatment

Sporozoites were prepared according to You et al., 1998 [15]. Briefly, after excystation, the *C. parvum* oocysts and sporozoite mixture was filtered through a 3.0 µM filter using a syringe and centrifuged, washed and counted. Isolated sporozoites were incubated with different SCFAs for 45 min, then washed several times to remove SCFAs before plating on cell monolayers. Sporozoites were then inoculated onto HCT-8 cells and incubated for 3 h. Monolayers were then washed, media was replaced and parasites were allowed to develop for 48 h, as described in the culture assay above. Cells were fixed and labeled and parasitic stages were quantitated as described above.

### 2.4. Measurement of Compound Toxicity for Host Cell Cultures

Test compound toxicity for HCT-8 cell cultures was assessed using the CellTiter 96 Non-Radioactive Cell Proliferation/Cytotoxicity assay as recommended by the manufacturer (Promega Corporation, Madison, WI, USA). Briefly, after cultured cells were incubated with compounds for 48 h, a tetrazolium dye solution was added and cultures were incubated for an additional 4 h to allow for the conversion of the assay substrate to the formazan product MTT [(3(4,5-dimethyl-thiazol-2-yl)]. A “stop solution” added to each sample solubilized the formazan product over a 1 h period at room temperature. The absorbance of the formazan product was measured at 570 nm using a 96-well plate reader. The cytotoxicity concentration (CC_50_) was defined as the concentration required to reduce cell viability by 50% compared with the untreated control.

### 2.5. Measurement of Cytokine Production in Response to Butyrate, C. parvum Infection or the Histone Deacetylase (HDAC) Inhibitor, PCI 34051

For the cytokine assay, the in vitro infection assay was performed as described above. Supernatants from 24-well plates were collected and stored at –80 °C. Luminex multiplex assays were completed by Eve Technologies using the Human Cytokine discovery Array 48-plex Panel (Calgary, AB, Canada).

### 2.6. Apoptosis Assay

In vitro assays were performed as described above. Briefly, HCT-8 cells were infected with *C. parvum* and/or treated with 1 mM butyrate for 24 h, after which the cells were analyzed for percent apoptosis. Control wells (uninfected, untreated) were compared with treated wells and infected wells. Apoptosis in HCT-8 cell cultures was measured using a commercially available assay (Promega Corporation, Madison, WI, USA). Caspase-Glo3/7 reagent was added directly to the assay wells. This results in cell lysis, followed by caspase cleavage of the DEVD substrate and the generation of luminescence. The peptide z-Val-Ala-Asp-fmk (z-VAD-fmk), a cell-permeant pan-caspase inhibitor that irreversibly binds to the catalytic site of caspase proteases and inhibits the induction of apoptosis (Promega, Madison, WI, USA), was used at a concentration of 20 μM for the inhibition of caspase, per the manufacturer’s recommendations. The amount of luminescence (proportional to the amount of apoptosis) is displayed as relative light units.

### 2.7. Autophagy Assay

In vitro assays were performed as described above. Briefly, HCT-8 cells were infected with *C. parvum* and/or treated with 1 mM butyrate for 24 h, after which the cells were analyzed for percent apoptosis. Control wells (uninfected, untreated) were compared with treated wells and infected wells. An autophagy assay was used following the recommendation of the manufacturer (Sigma-Aldrich, St. Louis, MO, USA). The medium was removed from the wells and 100 µL of the proprietary autophagosome detection reagent was added directly to each well. The plate was then incubated for 45 min at 37 °C and 5% CO_2_. The detection reagent was then removed, and each well was washed 3 times with the provided wash buffer before measuring fluorescence (λ_ex_ = 360/λ_em_ = 520 nm) with a microplate reader. The intensity of the fluorescence correlates directly with the amount of autophagy observed.

## 3. Results

### 3.1. Short-Chain Fatty Acids Inhibit Growth of C. parvum In Vitro

To examine the effect of SCFAs on the growth of the parasite, we infected HCT-8 cells with *C. parvum* and treated cells with either butyrate, propionate, acetate or valproate for a period of 48 h. As shown in Table 1, acetate, butyrate and propionate had activities in the 2–8 mM range (physiologically relevant concentrations), while valproate was less active with an EC_50_ of 10 mM. We also examined the effect of SCFAs on the extracellular stage of the parasite, the sporozoites (Table 1). Incubation time with SFCAs was limited to 45 min because of the short-term viability of sporozoites outside a host cell. However, this time frame should be sufficient to model the time between the excystation of oocysts in the gut and the invasion of epithelial cells. We found that there was no inhibition of sporozoites by acetate, propionate or valproate, while butyrate was inhibitory with an EC_50_ of 2 mM.

We then examined short-chain fatty acids in combination with one another, which would better simulate the environment of the intestinal tract. We chose to examine acetate, propionate and butyrate, which account for 90% of the short-chain acids in the gut. We found a slight increase in efficacy with an additive effect at a 3:1:1 (acetate:propionate:butyrate) concentration (Table 2). Additionally, cytotoxicity assays were performed using a tetrazolium-based assay to look for cellular viability. No toxicity was observed with treatment of either single or a combination of short-chain fatty acids at concentrations of less than 200 µM (Table 2). Because butyrate inhibited both the sporozoite stage and intracellular stages and more is known about possible butyrate mechanisms of action, subsequent studies were focused on it.

### 3.2. Butyrate and C. parvum Infection Increases Expression of IL-8 and IL-1a in HCT-8 Cells

Short-chain fatty acids are known histone deacetylase (HDAC) inhibitors. Butyrate, in particular, is reported to specifically inhibit HDAC8 and HDAC9 in keratinocytes [16]. Interestingly, this led to an increase in inflammatory response in these cells [16] and sebocytes [17]. To determine if butyrate and a more specific inhibitor of HDAC8 and HDAC9, PCI 34051, might generate a similar inflammatory response in HCT-8 cells, we evaluated the cytokine response after a 48 h treatment. Prior to that, we evaluated the anti-cryptosporidial activity of PCI 34,051 and two other broad spectrum HDAC inhibitors, trichostatin A and apicidin, which were previously reported to have anti-cryptosporidial activity at 30 ng/mL [18]. We found that apicidin and trichostatin A had EC_50_ of 20 and 40 nM, respectively, while PCI 34,051 had potent anti-cryptosporidial activity in the range of 1–7 nM, with no toxicity observed below 200 nM (Table 3).

**Figure 1:**
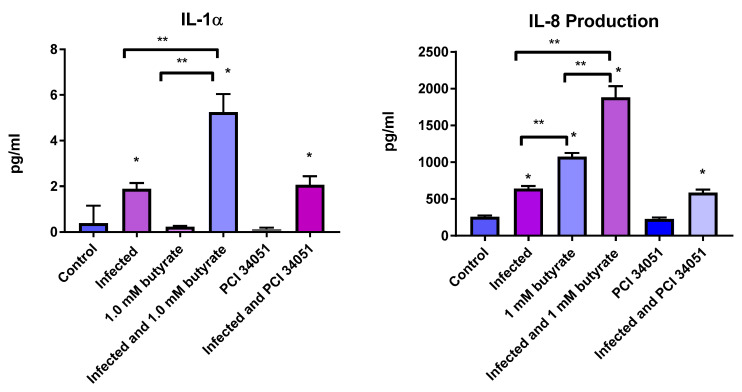
Effects of short-chain fatty acids on cytokine expression. HCT-8 cells were infected with *C. parvum* and/or treated with 1 mM butyrate for 48 h, after which supernatants were analyzed for cytokine production using the Luminex 100 system (Luminex). Amounts of cytokines, IL-1α (**left figure**) and IL-8 (**right figure**) are shown above. Values are reported as relative light units. Control wells (uninfected, untreated) were compared with treated and infected wells. Values are means ± SEM, *n* = 4. * Different from control, *p* < 0.05. ** Different from the experimental group as indicated by bar, *p* < 0.05.

We then compared cytokine responses in the presence of butyrate and with a known HDAC 8 inhibitor, PCI 34051. Overall, the majority of cytokines (including IL-6 and TNF-α, key inflammatory cytokines) in the 48-plex assay were not significantly altered by butyrate or PCI 34,051, either alone or in combination with infection. However, we did find significant increases in IL-8 and IL-1α in *C. parvum*-infected wells. Additionally, when cells were infected with *C. parvum* and then treated with butyrate and compared to butyrate treatment alone or *C. parvum* infection alone, a significant increase was observed over infection or butyrate treatment alone (Figure 1).

### 3.3. Butyrate and C. parvum Infection Increases Apoptosis in HCT-8 Cells

**Figure 2:**
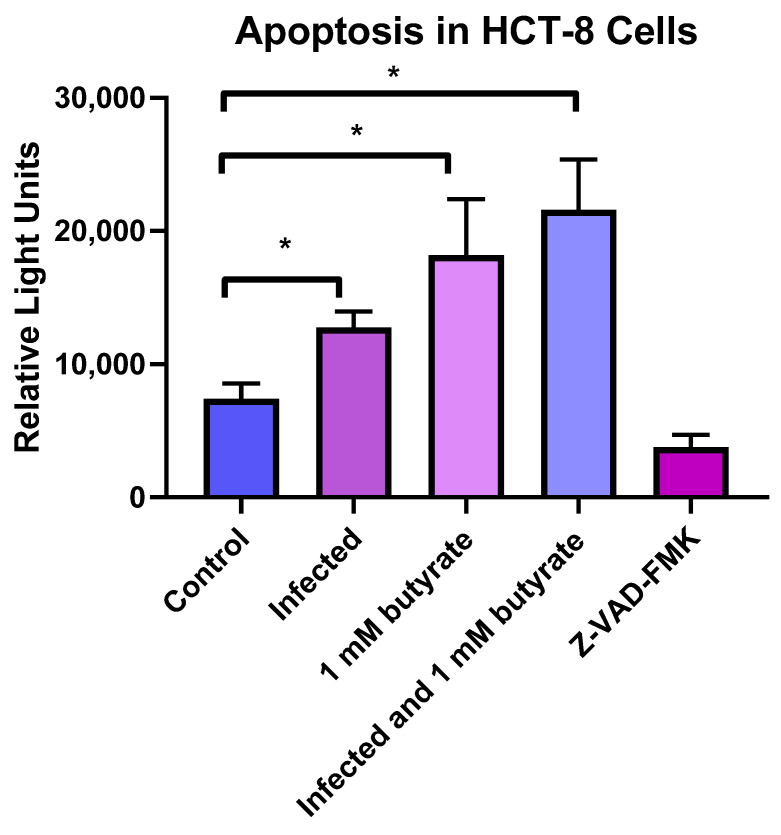
Amount of apoptosis in *C. parvum*-infected and butyrate-treated cells. HCT-8 cells were infected with *C. parvum* and/or treated with 1 mM of butyrate for 24 h, after which the cells were analyzed for percent apoptosis. Control wells (uninfected, untreated) were compared with treated wells and infected wells. The peptide Z-VAD-FMK, which inhibits apoptosis, served as a positive control. Values are means ± SEM, *n* = 4. * Different from control, *p* < 0.05.

Another possible mechanism of inhibition is through either apoptosis or autophagy. Butyrate, in particular, was reported to induce autophagy and apoptosis in colon cells in vitro [19]. We therefore looked at apoptosis and autophagy in HCT-8 cells following 24 h incubation with butyrate in the presence and absence of *C. parvum* infection. We found that *C. parvum* infection resulted in an increase in apoptosis, and treatment with 1.0 mM butyrate alone resulted in a significantly higher percentage of apoptosis (*p* < 0.05) when compared to control (Figure 2). The combination of *C. parvum* infection and butyrate treatment 3 h after infection resulted in an additional increase in apoptosis, which was also significantly different than the control (*p* < 0.05) and the infected alone group (*p* < 0.05), but, while increased, was not significantly different than the butyrate treatment alone.

### 3.4. Autophagy Is Decreased in HCT-8 Cells Infected with C. parvum and Treated with Butyrate

**Figure 3:**
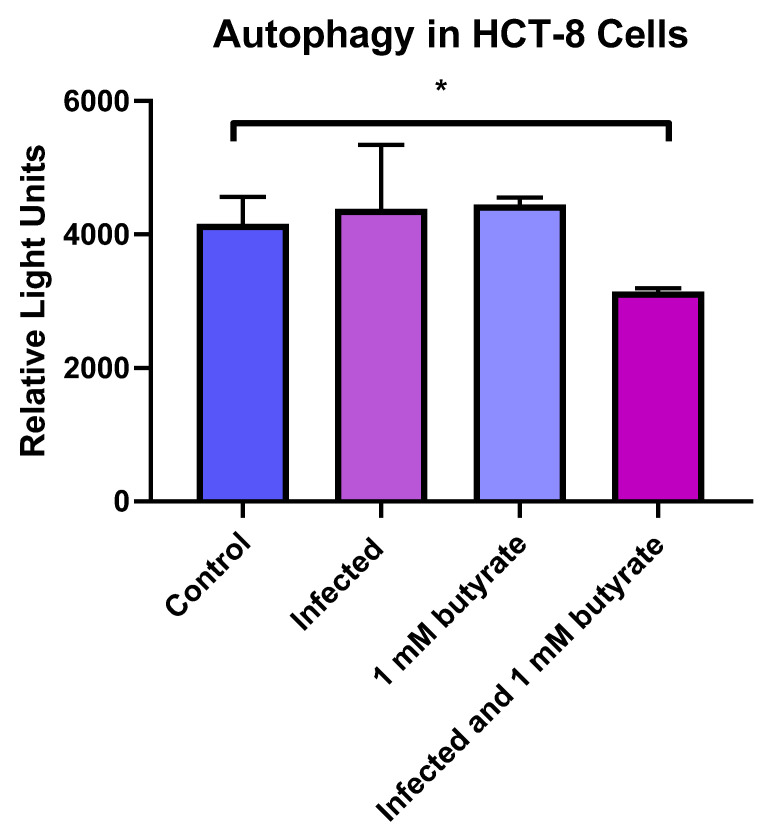
Amount of autophagy in *C. parvum*-infected and butyrate-treated cells. HCT-8 cells were infected with *C. parvum* and/or treated with 1 mM of butyrate for 24 h, after which the cells were analyzed for percent autophagy. Control wells (uninfected, untreated) were compared to treated control wells. Values are means ± SEM, *n* = 4. * Different from control, *p* < 0.05.

We also looked at the amount of autophagy in *C. parvum*-infected or butyrate-treated HCT-8 cells. As shown in Figure 3, we found in no difference in the infected or butyrate-treated cells alone. However, there was a significant decrease in autophagy in cells that were infected with *C. parvum* and treated with butyrate, which may relate to an overall increase in apoptosis when infection and treatment are combined.

## 4. Discussion

Short-chain fatty acids are important metabolites that provide fuel to epithelial cells, regulate gene expression and modulate the immune response. Changes in the microbiome due to drug or antibiotic treatment or malnutrition may lead to a depletion in SFCAs. The depletion of certain SFCAs may affect susceptibility to *C. parvum* infection or may have a beneficial effect on the growth of the parasite [7].

In addition to their effect on immune cells, SFCAs have been shown to have an effect on several protozoa, either directly or on the infected host cell. For example, acetate, butyrate and propionate inhibited encystment of *E. invadens* when incubated individually in a range of 6–22 mM [20]. Valproate, butyrate and 4-phenylbuyrate were reported to inhibit *T. gondii* in vitro [21]. More recently, treatment of mice with valproate was reported to reduce the number of *T. gondii* cysts in the brains of mice [22].

In the present study, we found that all of the SCFAs tested inhibited *C. parvum* with an EC_50_ between 1 and 8 mM, concentrations that are at physiological levels in the intestinal tract [23]. This is comparable to activity reported against *T. gondii*, which found IC_50_s in the range of 1–5 mM when cell monolayers were infected and then treated with either sodium butyrate or valproate [21]. Since all short-chain fatty acids had activity and are present in the lower small intestine, we examined the combination of the top 3 abundant SCFAs (accounting for 90% of the total SCFAs found in the intestinal tract) [24] on the inhibition of epithelial cells. Acetate is found at higher concentrations in the gut (4–10 times higher than butyrate or propionate [10]). When SCFAs were combined at a 3:1 ratio, there was an additive increase in efficacy.

Additionally, one SCFA, butyrate, inhibited the sporozoite stage of *C. parvum* with an EC_50_ of 2.0 mM. Since butyrate inhibited both the intracellular and the sporozoite stages, it is possible that either the host cell or the inhibition of parasitic histone deacetylases (HDACs) were partially responsible for the anti-cryptosporidial activity. HDACs play an important role in transcription and regulation, and at concentrations between 1 and 2 mM, butyrate and SCFAs can have a direct effect on histone deacetylases [25,26]. Additionally, inhibitors of histone deacetylase have been shown to decrease *C. parvum* growth in vitro [27].

Three class II HDAC enzymes have been identified in *C. parvum* [28]: CpHDAC1, CpHDAC2 and CpHDAC3. The latter, CpHDAC3, was cloned and expressed as a recombinant protein and evaluated against several HDAC inhibitors. Several inhibitors were found to have activity, including vorinostat, which had both in vitro activity and efficacy in a mouse model [29]. However, in this same study, butyrate did not inhibit CpHDAC3, suggesting that another CpHDAC was inhibited, or another target is responsible for the inhibitory activity we observed in our study.

Butyrate has also been shown to activate inflammatory signaling in keratinocytes, resulting in increased cytokine production. It has recently been reported that in keratinocytes, butyrate inhibited HDAC 8 and HDAC 9 specifically, and that this led to increased immune cytokine expression through the MAP2K3 pathway [16]. This led us to speculate if a specific inhibitor of HDAC8 or HDAC9 could inhibit *C parvum* parasitic growth and whether increased cytokines could be generated in response to butyrate or HDAC treatment. We chose to look at PCI 34051, a compound that more narrowly inhibits HDAC8 or HDAC9 and has been found to be active against the HDAC8 of the parasitic organism *S. mansoni* [30]. Interestingly, we found that PCI 34051, an HDAC8 inhibitor, was a potent inhibitor of *C. parvum* growth with an EC_50_ of 1 nM, more so than the more general HDAC inhibitors trichostatin A or apicidin.

We then examined whether similar cytokines could be generated in intestinal epithelial cells, as was described in keratinocytes in response to infection and butyrate or PCI 34051 treatment. We found that most cytokines were not altered by butyrate and *C. parvum* infection, including TNF-α and IL-6, key inflammatory cytokines found to be up-regulated in keratinocytes treated with butyrate and a toll receptor antagonist. However, IL-1α and IL-8 were significantly increased in response to *C. parvum* infection, which had been previously reported [31,32]. Moreover, there was an additional increase in IL-8 and IL-1α when cells were infected with *C. parvum* and then treated with butyrate versus butyrate treatment or *C. parvum* infection alone, suggesting that butyrate enhanced or boosted the expression of these cytokines in the presence of microbial stimulation.

The more specific inhibitor, PCI 34051, however, did not increase cytokine expression in HCT-8 cells as shown in keratinocytes. While we were unable to determine the mechanism of action of the drug in the present study, its potent anti-cryptosporidial activity merits further investigation.

Other mechanisms by which butyrate may deter pathogens or protect the host are through autophagy or apoptosis. We did not observe an increase in autophagy with either butyrate or *C. parvum* infection. In contrast, butyrate has been found to increase autophagy in other human colon cell lines [33,34]. In part, differences in results may be related to differences in methods and cell lines used in our study. Interestingly, we did find a significant decrease in autophagy and a simultaneous increase in apoptosis with cells that were both infected with *C. parvum* and treated with butyrate. Although increases/decreases in autophagy/apoptosis often correspond to one another, these mechanisms may have inverse effects. It has been noted that blockage of autophagy through chemical inhibitors can result in increases in apoptosis [19].

We did find that apoptosis significantly increased with both *C. parvum* infection, as previously reported [35], and with butyrate treatment. Apoptosis was further increased in host cells infected with C. *parvum* and treated with butyrate (significantly higher than control cells and infected cells), but not significantly higher than cells treated with butyrate alone.

In conclusion, acetate, propionate and butyrate have direct inhibitory activities against *C. parvum* in host cells, and butyrate can also affect sporozoite infectivity. Normal physiological levels of SCFAs, while not preventing infection, may help in keeping the infection low or in check. However, decreased SCFA levels (due to certain antibiotic treatments or other biological factors) may contribute to increased susceptibility and infection levels. Additionally, butyrate may contribute to inhibition through induction of apoptosis and priming the immune response, but future studies will help to further delineate the mechanism of the inhibition.

## Figures and Tables

**Table 1:** Effects of short-chain fatty acids on infected HCT-8 cells. EC_50_ and CC_50_ of HCT-8 cells infected and treated with different SCFAs.

SCFA	EC_50_ for Intracellular Stages	EC_50_ for Sporozoite Stages	Cytotoxicity Concentration (CC_50_) for HCT-8 Host Cells
Acetate	4.0 mM	>100 mM	>200 mM
Butyrate	3.0–8.0 mM	2.0 mM	>200 mM
Propionate	3.0–4.0 mM	>100 mM	>200 mM
Valproate	10.00 mM	>100 mM	>200 mM

**Table 2:** Effects of combination of short-chain fatty acids on infected HTC-8 cells. EC_50_ and CC_50_ of HCT-8 cells infected and treated with a combination of acetate, propionate and butyrate.

SCFA	EC_50_ for Intracellular Stages	Cytotoxicity Concentration (CC_50_) for HCT-8 Host Cells	Combination Index (CI) at EC_50_
Acetate:Propionate:Butyrate (3:1:1 ratio)	0.65 mM	>200 mM	1.01

**Table 3:** Effects of HDAC inhibitors on infected HCT-8 cells. EC_50_ and CC_50_ of HCT-8 cells infected and treated with different HDAC inhibitors.

HDAC Inhibitor	EC_50_ of Intracellular Stages	Cytotoxicity Concentration (CC_50_) for HCT-8 Host Cells
Trichostatin A	0.02 µM	>200 µM
Apicidin	0.04 µM	150 µM
PCI 34051	1–7 nM	>200 nM

## Data Availability

The data presented in this study are available on request from the corresponding author.

## References

[1] Shirley D.A., Moonah S.N., Kotloff K.L. (2012). Burden of disease from cryptosporidiosis. Curr. Opin. Infect. Dis..

[2] Kotloff K.L., Nataro J.P., Blackwelder W.C., Nasrin D., Farag T.H., Panchalingam S., Wu Y., Sow S.O., Sur D., Breiman R.F. (2013). Burden and aetiology of diarrhoeal disease in infants and young children in developing countries (the Global Enteric Multicenter Study, GEMS): A prospective, case-control study. Lancet.

[3] Checkley W., White A.C., Jaganath D., Arrowood M.J., Chalmers R.M., Chen X.M., Fayer R., Griffiths J.K., Guerrant R.L., Hedstrom L. (2015). A review of the global burden, novel diagnostics, therapeutics, and vaccine targets for *Cryptosporidium*. Lancet Infect. Dis..

[4] Chappell C.L., Darkoh C., Shimmin L., Farhana N., Kim D.K., Okhuysen P.C., Hixson J. (2016). Fecal indole as a biomarker of susceptibility to *Cryptosporidium* infection. Infect. Immun..

[5] Harp J.A., Chen W., Harmsen A.G. (1992). Resistance of severe combined immunodeficient mice to infection with *Cryptosporidium paruvm*: The importance of intestinal microflora. Infect. Immun..

[6] Sacco R.E., Haynes J.S., Harp J.A., Waters W.R., Wannemuehler M.J. (1998). *Cryptosporidium parvum* initiates inflammatory bowel disease in germfree T cell receptor-alpha-deficient mice. Am. J. Pathol..

[7] Charania R., Wade B.E., McNair N.N., Mead J.R. (2020). Changes in the microbiome of *Cryptosporidium*-infected mice correlate to differences in susceptibility and infection levels. Microorganisms.

[8] Mammeri M., Obregon D.A., Chevillot A., Polack B., Julien C., Pollet T., Cabezas-Cruz A., Adjou K.T. (2020). *Cryptosporidium parvum* infection depletes butyrate producer bacteria in goat kid microbiome. Front. Microbiol..

[9] Kim C.H. (2021). Control of lymphocyte functions by gut microbiota-derived short-chain fatty acids. Cell. Mol. Immunol..

[10] Parada Venegas D., De la Fuente M.K., Landskron G., Gonzalez M.J., Quera R., Dijkstra G., Harmsen H.J.M., Faber K.N., Hermoso M.A. (2019). Short Chain Fatty Acids (SCFAs)-mediated gut epithelial and immune regulation and its relevance for inflammatory bowel diseases. Front. Immunol..

[11] Tsuda H., Ochiai K., Suzuki N., Otsuka K. (2010). Butyrate, a bacterial metabolite, induces apoptosis and autophagic cell death in gingival epithelial cells. J. Periodontal Res..

[12] Mead J., McNair N. (2006). Antiparasitic activity of flavonoids and isoflavones against *Cryptosporidium parvum* and *Encephalitozoon intestinalis*. FEMS Microbiol. Lett..

[13] Geissmann Q. (2013). OpenCFU, a new free and open-source software to count cell colonies and other circular objects. PLoS ONE.

[14] Chou T.C., Talalay P. (1984). Quantitative analysis of dose-effect relationships: The combined effects of multiple drugs or enzyme inhibitors. Adv. Enzym. Regul..

[15] You X., Schinazi R.F., Arrowood M.J., Lejkowski M., Juodawlkis A.S., Mead J.R. (1998). In-vitro activities of paromomycin and lasalocid evaluated in combination against *Cryptosporidium parvum*. J. Antimicrob. Chemother..

[16] Sanford J.A., Zhang L.J., Williams M.R., Gangoiti J.A., Huang C.M., Gallo R.L. (2016). Inhibition of HDAC8 and HDAC9 by microbial short-chain fatty acids breaks immune tolerance of the epidermis to TLR ligands. Sci. Immunol..

[17] Sanford J.A., O’Neill A.M., Zouboulis C.C., Gallo R.L. (2019). Short-chain fatty acids from *Cutibacterium acnes* activate both a canonical and epigenetic inflammatory response in human sebocytes. J. Immunol..

[18] Darkin-Rattray S.J., Gurnett A.M., Myers R.W., Dulski P.M., Crumley T.M., Allocco J.J., Cannova C., Meinke P.T., Colletti S.L., Bednarek M.A. (1996). Apicidin: A novel antiprotozoal agent that inhibits parasite histone deacetylase. Proc. Natl. Acad. Sci. USA.

[19] Zhang J., Yi M., Zha L., Chen S., Li Z., Li C., Gong M., Deng H., Chu X., Chen J. (2016). Sodium butyrate induces endoplasmic reticulum stress and autophagy in colorectal cells: Implications for apoptosis. PLoS ONE.

[20] Byers J., Faigle W., Eichinger D. (2005). Colonic short-chain fatty acids inhibit encystation of *Entamoeba invadens*. Cell. Microbiol..

[21] Strobl J.S., Cassell M., Mitchell S.M., Reilly C.M., Lindsay D.S. (2007). Scriptaid and suberoylanilide hydroxamic acid are histone deacetylase inhibitors with potent anti-Toxoplasma gondii activity in vitro. J. Parasitol..

[22] Enshaeieh M., Saadatnia G., Babaie J., Golkar M., Choopani S., Sayyah M. (2021). Valproic acid inhibits chronic *Toxoplasma* infection and associated brain inflammation in mice. Antimicrob. Agents Chemother..

[23] Topping D.L., Clifton P.M. (2001). Short-chain fatty acids and human colonic function: Roles of resistant starch and nonstarch polysaccharides. Physiol. Rev..

[24] Rios-Covian D., Ruas-Madiedo P., Margolles A., Gueimonde M., de Los Reyes-Gavilan C.G., Salazar N. (2016). Intestinal short chain fatty acids and their link with diet and human health. Front. Microbiol..

[25] Andriamihaja M., Chaumontet C., Tome D., Blachier F. (2009). Butyrate metabolism in human colon carcinoma cells: Implications concerning its growth-inhibitory effect. J. Cell. Physiol..

[26] Markiewicz L.H., Ogrodowczyk A.M., Wiczkowski W., Wroblewska B. (2021). Phytate and butyrate differently influence the proliferation, apoptosis and survival pathways in human cancer and healthy colonocytes. Nutrients.

[27] Murakoshi F., Bando H., Sugi T., Adeyemi O.S., Nonaka M., Nakaya T., Kato K. (2020). Nullscript inhibits *Cryptosporidium* and *Toxoplasma* growth. Int. J. Parasitol. Drugs Drug Resist..

[28] Rider S.D., Zhu G. (2009). An apicomplexan ankyrin-repeat histone deacetylase with relatives in photosynthetic eukaryotes. Int. J. Parasitol..

[29] Guo F., Zhang H., Fritzler J.M., Rider S.D., Xiang L., McNair N.N., Mead J.R., Zhu G. (2014). Amelioration of *Cryptosporidium parvum* infection in vitro and in vivo by targeting parasite fatty acyl-coenzyme A synthetases. J. Infect. Dis..

[30] Stolfa D.A., Marek M., Lancelot J., Hauser A.T., Walter A., Leproult E., Melesina J., Rumpf T., Wurtz J.M., Cavarelli J. (2014). Molecular basis for the antiparasitic activity of a mercaptoacetamide derivative that inhibits histone deacetylase 8 (HDAC8) from the human pathogen *Schistosoma mansoni*. J. Mol. Biol..

[31] Laurent F., Eckmann L., Savidge T.C., Morgan G., Theodos C., Naciri M., Kagnoff M.F. (1997). *Cryptosporidium parvum* infection of human intestinal epithelial cells induces the polarized secretion of C-X-C chemokines. Infect. Immun..

[32] Maillot C., Gargala G., Delaunay A., Ducrotte P., Brasseur P., Ballet J.J., Favennec L. (2000). Cryptosporidium parvum infection stimulates the secretion of TGF-beta, IL-8 and RANTES by Caco-2 cell line. Parasitol. Res..

[33] Priyamvada S., Jayawardena D., Bhalala J., Kumar A., Anbazhagan A.N., Alrefai W.A., Borthakur A., Dudeja P.K. (2021). Cryptosporidium parvum infection induces autophagy in intestinal epithelial cells. Cell. Microbiol..

[34] Horikoshi M., Pasquali L., Wiltshire S., Huyghe J.R., Mahajan A., Asimit J.L., Ferreira T., Locke A.E., Robertson N.R., Wang X. (2016). Transancestral fine-mapping of four type 2 diabetes susceptibility loci highlights potential causal regulatory mechanisms. Hum. Mol. Genet..

[35] Chen X.M., Gores G.J., Paya C.V., LaRusso N.F. (1999). *Cryptosporidium parvum* induces apoptosis in biliary epithelia by a Fas/Fas ligand-dependent mechanism. Am. J. Physiol.-Gastrointest. Liver Physiol..

